# Magnitude and Determinants of Dental Anxiety among Adult Patients Attending Public Dental Clinics in Dar-Es-Salaam, Tanzania

**DOI:** 10.1155/2021/9965204

**Published:** 2021-05-08

**Authors:** Kauther Musalam, Karpal S. Sohal, Sira S. Owibingire, Baraka Kileo

**Affiliations:** ^1^Department of Dental Services, Muhimbili National Hospital, P. O. Box 65000, Dar es Salaam, Tanzania; ^2^Department of Oral and Maxillofacial Surgery, School of Dentistry, Muhimbili University of Health and Allied Sciences, P. O. Box 65014, Dar es Salaam, Tanzania

## Abstract

**Introduction:**

It is estimated that, about 40% of the population suffer from dental anxiety. Dental anxiety is considered to be complex and multifactorial with a wide range of provoking factors which may be patient, provider, or environment-related.

**Aim:**

This study aimed to assess the magnitude and determinants of dental anxiety among adult patients attending public dental clinics in Dar es Salaam, Tanzania. *Methodology*. This was a descriptive cross-sectional study carried out in 4 public hospitals in Dar es Salaam, Tanzania. It included 300 adult patients who had dental caries, periodontal diseases, or dental trauma. Data were collected using a self-administered Modified Dental Anxiety Scale (MDAS) questionnaire. Data were analyzed using the SPSS computer software version 23. A one-way Analysis of Variance (ANOVA) was used to assess the association between variables, and the significance level was set at *p* < 0.05.

**Results:**

The mean age of participants was 32.18 years (±11.06 SD) with a male-to-female ratio of 1 : 1.43. The means MDAS score was 12.84 ± 4.99. Tooth extraction had the highest mean MDAS score. The majority (261, 87%) of participants had mild-to-severe anxiety. The most common (72.2%) anxiety-provoking factor was an unsympathetic dentist; others included unawareness of the procedure to be carried out (58.3%) and the presence of apprehensive patients (52.0%). The level of anxiety was found to be statistically significantly associated (*p* < 0.05) with young age [*p*=0.009, AOR 3.06 (95% CI, 1.32, 7.09), female patients [*p* < 0.001, AOR 4.45 (95% CI, 2.05, 9.70)], and a higher education level [*p* < 0.05, AOR 2.32 (95% CI, 1.03, 5.25)].

**Conclusion:**

The prevalence of dental anxiety was high among the participants of this study. Female gender, young age, and a higher level of education constituted determinants of dental anxiety. An unsympathetic practitioner, unawareness of the procedure, and presence of apprehensive patients were the common anxiety-provoking factors.

## 1. Introduction

Dental anxiety afflicts a significant proportion of people of all ages from different social classes [1, 2]. It remains to be of concern for both the dental practitioners and the patients [1] since it often results in inadequate oral health by complete avoidance of dental treatment, irregular dental attendance, or poor cooperation [2, 3].

In one study among the ten most commonly feared situations, dental anxiety ranked the fourth next to fear of snakes, height, and physical injuries [4]. It is estimated to affect approximately 36% of the population, of whom 10 to 20% suffer from extreme dental anxiety [5, 6]. Dental anxiety is influenced by sociodemographic characteristics of patients such as age, gender, marital status, level of education, income, and place of residence [7]. It has been reported that young, single, and female patients are usually more anxious when compared to their counterparts, respectively [8].

Dental anxiety is considered to be complex and multifactorial with a wide range of provoking factors which may be patient-, provider-, or environment-related [5]. The patient-related causes include past dental experience, pain, the influence of family and peer experience, and personality, whereas provider-related causes include communication techniques and provider's bad behavior [5, 9, 10]. The environmental-related causes include the sound of drills, presence of apprehensive patients in the clinic, unpleasant smell around the clinic area, longer waiting period, and the sight of blood [5, 9, 10].

A previous study from Tanzania [11] that was carried out almost a decade ago found that only 21.7% of the participants had dental anxiety. Another study reported that the prevalence of dental anxiety in primary school teachers was 1.2% [12]. A study to assess dental anxiety among undergraduate students in a medical university in Tanzania found that the prevalence of anxiety was 55% [13].

In the past decade, the field of dentistry has changed significantly in Tanzania. There are younger and more competent practitioners who employ the latest technological advancement in the field. Despite these advancements, the patients still seek treatment late with unrestorable teeth, and thus extraction is the most common treatment of dental-related problems [14].

The previous studies carried out in Tanzania did not assess the impact of tooth extraction on causing dental anxiety. Taking this into account, this study aimed to assess the magnitude and determinants of dental anxiety among adult patients attending public dental clinics in Dar es Salaam, Tanzania., by incorporating the aspect of tooth extraction. The results from this study thus give sight on the level of dental anxiety among populations and factors associated with dental anxiety, therefore, providing the necessary information to the dental practitioner on how best to handle patients and plan their treatment accordingly.

## 2. Materials and Methods

This was a descriptive cross-sectional study carried out in public hospitals in Dar es Salaam between October 2019 and March 2020. The study included Muhimbili National Hospital, Muhimbili University of Health and Allied Sciences (MUHAS) Dental Clinic, and two regional referral hospitals (Amana and Mwananyamala) in Dar es Salaam, Tanzania.

The inclusion criteria included dental patients aged 18 and above, patients with dental caries, periodontal disease, or dental trauma. And the exclusion criteria included all those patients who were not mentally sound, dental patients with maxillofacial bone fractures, and orofacial neoplasms.

The sample size was estimated using the population adjustment formula for single proportion estimation [15] based on a 95% confidence level, a precision of 5%, and power of 0.8 with an expected proportion of 22% based on the previous study [11] yielding a total sample of 300 participants. A stratified random sampling method was used to obtain the participants. The included hospitals were used as strata and the simple random sampling method was used for the dental clinic to attain the sample size required (300 participants). Due to the variation in the number of dental patients who visited different dental clinics in the city, dental patients attending MUHAS Dental Clinic contributed 35% of the estimated sample size. Those who attended MNH Dental Clinic comprised 25% and those patients who attended Mwananyamala and Amana Hospitals Dental Clinics constituted 20% each.

Patients who met the criteria to participate in the study were interviewed by the same researcher using a predesigned questionnaire in a separate room while they were waiting to be attended/treated (pretreatment). The questionnaire was translated from English to Swahili language, and it was pretested in 30 pilot patients (10% of the estimated sample size) before using it for the study. It is composed of questions on the sociodemographic characteristics, 5 sets of questions from the Modified Dental Anxiety Scale (MDAS) each question scoring 1 to 5, with 1 being not anxious and 5 being extremely anxious. An additional question on the stimuli associated with dental anxiety and a question regarding tooth extraction were also added to the MDAS set of questions. The six questions were summed together to produce a total score ranging from 6 to 30.

The data obtained from this study were coded and entered into the computer program and analyzed using SPSS software version 23.0. Data was presented in the form of the mean for continuous variables and percentages for categorical variables.

The age of the patients was dichotomized into <40 years and ≥40 years. The level of education for the participants was categorized into those with low level (no formal and primary education) and high level (secondary and tertiary education). Marital status was grouped into those living with partners (married, cohabiting) and those living without partners (single, divorced, widowed). The residence was divided into urban areas (urban center) and suburban areas. Employment status was grouped into those with informal employment, public formal employment, and private formal employment. Since each question has five scores ranging from “not anxious” to “extremely anxious,” in an ascending order from 1 to 5, each question thus caries a possible maximum score of 5 with a total possible maximum score of 30 for the entire scale with higher scores indicative of greater dental fear. A cutoff score of >19 was used to identify individuals with high levels of dental anxiety. Thus, the level of anxiety was classified according to the total score obtained as no anxiety (total score 6), mild-to-moderate anxiety (7–19), severe-to-extreme anxiety (20–30).

The data was presented using frequencies and percentages in the form of tables and charts. A one-way Analysis of Variance (ANOVA) was used to assess the differences in dental anxiety for selected factors. The probability level of *p* < 0.05 was selected for statistical significance. Univariate and multivariate linear and logistic regression models were used to assess associations between the sociodemographic characteristic of participants and dental anxiety.

Ethical clearance was sought from the MUHAS Institution Review Board, and permission to conduct the study was obtained from the appropriate authorities of different departments of MUHAS Dental Clinic, MNH, Amana, and Mwananyamala Hospitals. Only those participants who freely gave consent to participate were included in the study. All information was handled confidentially and refusal to participate or withdraw from the study did not result in any consequence on the side of the patient.

## 3. Results

In this study, there were 300 participants. Their ages ranged from 18 years to 90 years with a mean age of 32.18 years (±11.06 SD). Females were 177 (59%) and the male-to-female ratio was 1 : 1.43. Most participants had a partner (163, 54.3%) and were in informal employment (129, 43.0%). The majority of the participants resided in suburban areas (231, 77.0%) and had a high level of education (250, 83.3%) (Table 1).

The MDAS score of participants ranged from 6 to 30, with a mean score of 12.84 ± 4.99. The overall mean score for each question of MDAS is represented in Table 2. Tooth extraction had the highest mean MDAS score while the prospect of a future visit to the dentist had the least mean MDAS score (Table 2).

The mean MDAS scores (overall and for specific items) of females, individuals aged less than 40 years, and those with higher education levels were more than those of males, individuals aged ≥ 40 years, and those with lower education levels, respectively. The observed difference in MDAS score within sex, age groups, and education levels was statistically significant (Tables 3 and 4 ).

The majority (261, 87%) of participants had mild-to-severe anxiety. Two hundred and forty (80%) participants had mild-to-moderate levels of anxiety followed by those with no anxiety (39, 13%) and very few (21, 7%) had severe/extreme anxiety. The association between age, sex, and education level of the participants and the level of anxiety were statistically significant (Table 5.) A multiple regression analysis was done for the associated factors. The model included all factors with a *p* value of <0.1 and an adjusted odds ratio (AOR) was calculated. The odds of the female patients being anxious were 4 folds higher than males (*p* < 0.001, AOR 4.45 (95% CI, 2.05, 9.70)). Young patients (<40 years) were 3 times more anxious than the older ones (*p*=0.009, AOR 3.06 (95% CI, 1.32, 7.09)), and participants with a high level of educations were 2 times more likely to be anxious compared to the those with a low level of education (*p* < 0.043, AOR 2.32 (95% CI, 1.03, 5.25)).

**Figure 1:**
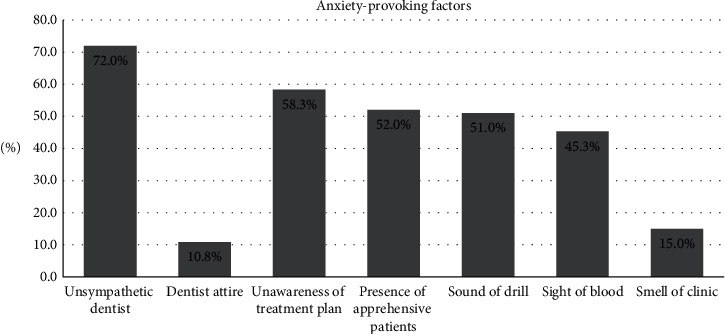
Percentage distribution of dental anxiety-provoking factors in clinical setting according to the response of participants.

Regarding the anxiety-provoking factors, unsympathetic dentist (281, 72.2%) was the most common provoking factor while the least (45, 15.0%) anxiety triggering factor was the smell of the clinic (Figure 1). There was no statistically significant association between the sociodemographics of the participant and different dental anxiety factors except for a few. Sex of the participants was significantly associated with unawareness of the procedure, presence of apprehensive patients, and sound of the drill while education level was significantly associated with unawareness of the dental procedure/plan to be carried out.

The odds of female participants being anxious due to unawareness of the treatment plan was nearly 2 folds higher than their male counterparts (*p* < 0.025, OR 1.64 (95% CI, 1.03, 2.62)). The presence of apprehensive patients in the clinic triggered dental anxiety in female participants by almost 3 folds more than in males (*p* < 0.001, OR 2.75 (95% CI, 1.71, 4.42)), whereas the sound of the drill triggered anxiety in female participants 2 times more than in males (*p*=0.001, OR 2.15 (95% CI, 1.35, 3.44)). The odds of participants with a high level of education to be anxious due to unawareness of dental treatment plan was nearly 2 folds higher than those with a low level of education (*p* < 0.026, OR 2.01 (95% CI, 1.09, 3.71)).

## 4. Discussion

Anxiety is a natural human emotion encountered in various situations including in dental practice, and it is a common reason for people avoiding dental treatment [16]. Dental anxiety can be triggered by provider-related factors and environment-related factors apart from personal factors [5, 9, 10]. It is important to assess levels and triggers of dental anxiety to manage the patient well. Since it is within the capacity of a dental practitioner to reduce the environmental and practitioners related factors, this study aimed to assess the level of anxiety in relation to the sociodemographic characteristics of participants and to evaluate the clinic related factors that influence dental anxiety among adult patients attending public dental clinics in Dar es Salaam, Tanzania.

The MDAS was used in this study because it is a simple, valid, and good predictor of patients' distress in the dental operatory [17, 18]. An additional question on the stimuli associated with dental anxiety and a question regarding tooth extraction was added to the MDAS set of questions because a significant proportion of the population views tooth extraction services as the first line and the desirable modality of treatment for toothaches [19].

Findings of the present study indicate that there was a high prevalence of dental anxiety among the participants similar to a report from a study done in India [20] contrary to the previous reports from Tanzania [11] and elsewhere [21–24]. The differences in the prevalence of anxiety between the current study and others may be attributed to the different research methodologies used, type of study setting, and characteristics of the study population.

Dental anxiety is typically related to demographic variables such as sex, age, and education [6]; similarly, in this study, the association between age, sex, and education level of the participants and the level of anxiety were statistically significant. According to the findings in the literature, dental anxiety is more common in females [23–25]. Similar results were obtained in our study whereby the odds of dental anxiety in females was 4 times more than in males. Most authors believe anxiety is more common in females because women tend to easily express their feelings of fear [20, 23–25]. Besides, it has been suggested that genetic factors and female reproductive hormones may play important roles in the expression of disorders like phobia, anxiety (including dental anxiety), panic, depression, stress, and fear in females [26].

Several studies have suggested that dental anxiety is lower in the older age groups [3, 21, 27, 28], and results from this study have similar findings. Young patients were 3 times more anxious than the older ones. Low levels of anxiety in older age may be because the aging process itself is characterized by a general decline in anxiety due to increased exposures over time which in turn allows one to develop tolerance to treatment [3, 21].

Contrary to the findings in the literature [1, 21], in this study, it was found that participants with a higher level of education were more anxious than those with lower education levels. Our findings were similar to the findings from elsewhere [8, 27, 29]. It could be argued that educated people cope better and rationalize a situation rather than avoiding it [1]; however, in our situation, it was not the case. The findings obtained in this particular study may be explained to be due to a lack of a culture of frequent dental visits in our locality. Thus, due to little awareness regarding dental health, the educated unlike their counterparts tend to search about their symptoms and possible treatment options on the Internet. In doing so, it creates a sense of anxiety in them because of what they read.

In the current study, it was depicted that participants would be more anxious when they were to have a tooth extracted, followed by when they were about to be injected with local anesthesia and have a tooth drilled. Similar results have been reported from different studies carried out elsewhere [1, 12, 21, 30, 31]. These findings indicate that invasive stimuli like extraction of a tooth tend to be most anxiety-provoking. This may be explained by the fact that invasive procedures are often associated with pain which has a vicious relation with anxiety.

Regarding the anxiety-provoking factors in this study, an unsympathetic practitioner was the most common provoking factor. Patients tend to feel more comfortable when a dentist is encouraging, caring, and sympathetic; thus, they tend to be relaxed and less anxious. Unsurprisingly, the smell of the clinic was the least anxiety-provoking stimuli in this study, and this may be attributed to the efforts made by the administration of public clinics in making sure the clinical environment is always clean, and chemicals used for disinfecting the surfaces have a pleasant smell. Smell plays an important role in transferring information about the surrounding environments, as such, unpleasant odor usually is associated with uneasiness, therefore, leading to anxiety [32].

From the current study, it was shown that unawareness of the procedure caused more anxiety in female patients and those with higher education levels by at least 2 folds, respectively. Being unaware of what is going to be done and what is the outcome of the procedure tends to make one feel as if they have no control over the situation. The sense of loss of control over the treatment procedure is a significant cause of anxiety [33].

In view of findings from this study, dental practitioners should strive to establish and maintain a good patient-dentist relationship and suitable communication with the patients. They should provide a sense of control to their patients by informing them of details of treatment in a simple and unthreatening manner. When managing patients with dental anxiety, the practitioner should follow a gentle, supportive, professional, sympathetic, and more considerate approach [34]. Moreover, when discussing the treatment plan, the practitioner should convey confidence in presenting it to the patient as this may serve to lessen negative evaluations from the patients [3], hence reducing anxiety. With low levels of anxiety, patients will not avoid dental treatment and will have regular dental attendance and good cooperation [2, 3].

Some limitations of this study include that only MDAS was used to evaluate their dental anxiety, the personal anxiety-provoking factors were not assessed, and the study was carried out in the hospital settings. Despite these limitations, the strengths of the study are based on the fact that an acceptable and well-recognized tool (MDAS questionnaire) was used, the participants were selected from four public hospitals, and the days of visit for data collection were randomly selected; moreover, the participants were also included randomly, thus reducing the bias.

## 5. Conclusion

The prevalence of dental anxiety was high among the participants of this study. Females, young patients, and those with higher levels of education were more anxious than their counterparts. An unsympathetic practitioner, unawareness of the procedure, presence of apprehensive patients, and sound of the drill were the common anxiety-provoking factors among the participants in the clinical environment.

## Figures and Tables

**Table 1:** Distribution of study participants according to sociodemographic characteristics.

Sociodemographic characteristics	Participants		
	Male (*n* = 123)	Female (*n* = 177)	Total (*n* = 300)
Age groups (years)			
<40	106 (86.2%)	136 (76.8%)	242 (80.7%)
40+	17 (13.8%)	41 (23.2%)	58 (19.3%)

Marital status			
With partner	58 (47.2%)	79 (44.6%)	137 (45.7%)
Without partner	65 (52.8%)	98 (55.4%)	163 (54.3%)

Residence			
Urban	27 (22.0%)	42 (23.7%)	69 (23.0%)
Suburban	96 (78.0%)	135 (76.3%)	231 (77.0%)

Education			
Low level	26 (21.1%)	24 (13.6%)	50 (16.7%)
High level	97 (78.9%)	153 (86.4%)	250 (83.3%)

Occupation			
Informal employment	42 (34.1%)	87 (49.2%)	129 (43.0%)
Employment (public)	24 (19.5%)	34 (19.2%)	58 (19.3%)
Employment (private)	57 (46.3%)	56 (31.6%)	69 (23.0%)

**Table 2:** Means and standard deviation of Modified Dental Anxiety Scale items.

MDAS item	Mean ± SD
Q1: if you were sitting in the waiting room	1.82 ± 0.96
Q2: if you were about to have a tooth drilled	2.45 ± 1.21
Q3: if you were about to have your teeth scaled and polished	1.90 ± 1.07
Q4: if you were about to have a local anesthetic injection into your gum	2.43 ± 1.19
Q5: if you were about to have a tooth extracted	2.68 ± 1.28
Q6: if you went to the dentist for treatment in the future	1.56 ± 0.97

**Table 3:** Overall mean MDAS score according to social-demographic characteristics of the participants.

Sociodemographic characteristics	Mean MDAS score
	Mean ± SD
Sex	
Male	1.62 ± 0.96
Female	1.96 ± 0.94
*p* value	0.001

Age groups (years)	
<40	13.31 ± 5.09
40+	10.83 ± 3.96
*p* value	0.001

Residence	
Urban	13.51 ± 5.29
Suburban	12.63 ± 4.89
*p* value	0.201

Education	
Low level	11.50 ± 4.97
High level	13.10 ± 4.96
*p* value	0.038

Occupation	
Informal employment	13.43 ± 4.66
Employment (public)	12.02 ± 4.42
Employment (private)	12.57 ± 5.55
*p* value	0.154

Marital status	
With partner	12.47 ± 4.82
Without partner	13.13 ± 5.12
*p* value	0.254

**Table 4:** MDAS score for each item according to social-demographic characteristics of the participants.

Sociodemographic characteristics	Mean MDAS score for each item					
	Q1 mean ± SD	Q2 mean ± SD	Q3 mean ± SD	Q4 mean ± SD	Q5 mean ± SD	Q6 mean ± SD
Sex						
Male	1.62 ± 0.96	2.15 ± 1.2	1.65 ± 1.01	2.07 ± 1.13	2.34 ± 1.23	1.54 ± 1.00
Female	1.96 ± 0.94	2.66 ± 1.17	2.07 ± 1.08	2.68 ± 1.17	2.91 ± 1.23	1.57 ± 0.95
*p* value	0.002	<0.001	0.001	<0.001	<0.001	0.766

Age groups (years)						
<40	1.87 ± 1.01	2.56 ± 1.22	1.94 ± 1.11	2.55 ± 1.21	2.80 ± 1.29	1.60 ± 1.01
40+	1.60 ± 0.70	2.00 ± 1.06	1.72 ± 0.85	1.93 ± 0.99	2.17 ± 1.08	1.40 ± 0.75
*p* value	0.056	0.001	0.172	<0.001	0.001	0.163

Residence						
Urban	1.84 ± 1.02	2.55 ± 1.26	2.07 ± 1.13	2.62 ± 1.19	2.88 ± 1.27	1.54 ± 0.93
Suburban	1.81 ± 0.94	2.42 ± 1.19	1.84 ± 1.05	2.37 ± 1.19	2.61 ± 1.27	1.56 ± 0.98
*p* value	0.840	0.446	0.120	0.125	0.124	0.843

Education						
Low level	1.64 ± 0.92	2.22 ± 1.30	1.72 ± 1.05	2.16 ± 1.08	2.26 ± 1.10	1.50 ± 0.91
High level	1.86 ± 0.97	2.50 ± 1.19	1.93 ± 1.07	2.48 ± 1.21	2.76 ± 1.29	1.57 ± 0.97
*p* value	0.147	0.135	0.201	0.08	0.01	0.652

Occupation						
Informal employment	1.88 ± 0.97	2.65 ± 1.20	1.96 ± 1.11	2.52 ± 1.21	2.84 ± 1.29	1.57 ± 0.96 0.961.69 ±
Employment (public)	1.69 ± 0.78	2.26 ± 1.08	1.90 ± 0.95	2.24 ± 1.13	2.57 ± 1.24	1.36 ± 0.74
Employment (private)	1.81 ± 1.04	2.33 ± 1.26	1.82 ± 1.09	2.42 ± 1.19	2.54 ± 1.27	1.64 ± 1.08
*p* value	0.442	0.045	0.606	0.336	0.139	0.208

Marital status						
With partner	1.77 ± 0.93	2.31 ± 1.13	1.88 ± 1.01	2.34 ± 1.15	2.61 ± 1.24	1.55 ± 0.92
Without partner	1.86 ± 0.99	2.57 ± 1.26	1.91 ± 1.11	2.51 ± 1.22	2.73 ± 1.31	1.56 ± 1.01
*p* value	0.446	0.067	0.842	0.209	0.430	0.975

**Table 5:** Level of anxiety according to social-demographic characteristics of the participants.

Sociodemographic characteristics	Level of anxiety			*p* value
	None (score ≤6) *n* = 39	Mild/moderate (score 7–19) *n* = 240	Severe/extreme (score ≥20) *n* = 21	
Sex				
Male	27 (22.0%)	88 (71.5%)	8 (6.5%)	0.001
Female	12 (6.8%)	152 (85.9%)	13 (7.3%)	

Age groups (years)				
<40	25 (10.3%)	196 (81.0%)	21 (8.7%)	0.002
40+	14 (24.1%)	44 (75.9%)	−	

Residence				
Urban	9 (13.0%)	52 (75.4%)	8 (11.6%)	0.230
Suburban	30 (13.0%)	188 (81.4%)	13 (5.6%)	

Education				
Low level	14 (28.0%)	32 (64.0%)	4 (8.0%)	0.002
High level	25 (10.0%)	208 (83.2%)	17 (6.8%)	

Occupation				
Informal employment	10 (7.7%)	110 (85.3%)	9 (7.0%)	0.121
Employment (public)	10 (17.2%)	46 (79.3%)	2 (3.4%)	
Employment (private)	19 (16.8%)	84 (74.3%)	10 (8.8%)	

Marital status				
With partner	20 (14.6%)	110 (80.3%)	7 (5.1%)	0.409
Without partner	19 (11.7%)	130 (79.7%)	14 (8.6%)	

## Data Availability

The data used to support the findings of this study are freely available from the corresponding author on reasonable request.

## References

[1] Appukuttan D., Subramanian S., Tadepalli A., Damodaran L. (2015). Dental anxiety among adults: an epidemiological study in South India. *North American Journal of Medical Sciences*.

[2] Tunc E. P., Firat D., Onur O. D., Sar V. (2005). Reliability and validity of the modified dental anxiety scale (MDAS) in a Turkish population. *Community Dentistry and Oral Epidemiology*.

[3] Caltabiano M. L., Croker F., Page L. (2018). Dental anxiety in patients attending a student dental clinic. *BMC Oral Health*.

[4] Oosterink F. M. D., De Jongh A., Hoogstraten J. (2009). Prevalence of dental fear and phobia relative to other fear and phobia subtypes. *European Journal of Oral Sciences*.

[5] Beaton L., Freeman R., Humphris G. (2014). Why are people afraid of the dentist? Observations and explanations. *Medical Principles and Practice*.

[6] Milgrom P., Newton J. T., Boyle C., Heaton L. J., Donaldson N. (2010). The effects of dental anxiety and irregular attendance on referral for dental treatment under sedation within the National Health Service in London. *Community Dentistry and Oral Epidemiology*.

[7] Appukuttan D. P., Tadepalli A., Cholan P. K., Subramanian S. (2012). Prevalence of dental anxiety among patients attending a dental educational institution in Chennai, India–a questionnaire based study. *Oral Health Diagnostic Medicine*.

[8] Egbor P. E., Akpata O. (2014). An evaluation of the sociodemographic determinants of dental anxiety in patients scheduled for intra-alveolar extraction. *Libyan Journal of Medicine*.

[9] Hmud R., Walsh L. J. (2008). Dental anxiety: causes, complications and management approaches. *International Dentistry SA*.

[10] Minja I. K., Kahabuka F. K. (2019). *Dental Anxiety and its Consequences to Oral Health Care Attendance and Delivery. Anxiety Disord-from Child to Adulthood*.

[11] Mwimanzi P., Kahabuka F. K. (2011). Dental fear and associated factors among adults in Dar es Salaam, Tanzania. *Tanzania Dental Journal*.

[12] Minja I. K., Jovin A. C., Mandari G. J. (2016). Prevalence and factors associated with dental anxiety among primary school teachers in Ngara district, Tanzania. *Tanzania Journal of Health Research*.

[13] Laizer P., Nderkero T., Sohal K. (2018). Prevalence of dental anxiety among undergraduate students at Muhimbili university of health and allied Sciences, Tanzania. *International Journal of Psychosocial Rehabilitation*.

[14] Nyamuryekung’e K. K., Lahti S. M., Tuominen R. J. (2015). The relative patient costs and availability of dental services, materials and equipment in public oral care facilities in Tanzania. *BMC Oral Health*.

[15] Charan J., Biswas T. (2013). How to calculate sample size for different study designs in medical research?. *Indian Journal of Psychological Medicine*.

[16] Giri J., Pokharel P. R., Gyawali R., Bhattarai B. (2017). Translation and validation of modified dental anxiety scale: the Nepali version. *International Scholarly Research Notices*.

[17] Arslan S., Ertaş E. T., Ülker M. (2011). The relationship between dental fear and sociodemographic variables. *Erciyes Tip Dergisi*.

[18] Amir A., Kamate S., Gupta P. (2018). Assessment of dental anxiety using MDAS ( modified dental anxiety scale) among students in bareilly city-a cross sectional study section. *International Journal of Contemporary Medical Research*.

[19] Nyamuryekung’e K. K., Lahti S. M., Tuominen R. J. (2018). Attitudes towards tooth fillings in Tanzanian adults and its association with previous filling experience. *BMC Oral Health*.

[20] Sinha E., Rekha R., Nagashree S. (2019). Anxiety of dental treatment among patients visiting primary health centers. *Journal of Indian Association of Public Health Dentistry*.

[21] Bashiru B., Omotola O. (2016). Prevalence and determinants of dental anxiety among adult population in Benin City, Nigeria. *European Journal of General Dentistry*.

[22] Malvania E. A., Ajithkrishnan C. G. (2011). Prevalence and socio-demographic correlates of dental anxiety among a group of adult patients attending a dental institution in Vadodara city, Gujarat, India. *Indian Journal of Dental Research*.

[23] Saatchi M., Abtahi M., Mohammadi G., Mirdamadi M, Binandeh E. S (2015). The prevalence of dental anxiety and fear in patients referred to Isfahan Dental School, Iran. *Dental Research Journal*.

[24] Alhamed S. A., Halawani R. T., Ahmed H. A. (2019). Dental anxiety among adult patients attending public dental clinics in jeddah, Saudi arabia. *Current Science International*.

[25] Murad M., Ingle N., Assery M. (2020). Evaluating factors associated with fear and anxiety to dental treatment-A systematic review. *Journal of Family Medicine and Primary Care*.

[26] Kinrys G., Wygant L. E. (2005). Anxiety disorders in women: does gender matter to treatment?. *Revista Brasileira de Psiquiatria*.

[27] Faisal S., Zehra N., Hussain M. (2015). Dental anxiety among patients attending public and private dental hospitals of Karachi. *Journal of Pakistan Dental Association*.

[28] Nahla K. I., Al-Jdani M., Al-Aamoudi N., Sukkar S. (2016). Anxiety due to dental procedures and treatment among adult patients attending outpatient clinics in King. *Indian Journal of Medical Research*.

[29] Fotedar S., Bhardwaj V., Fotedar V. (2016). Dental anxiety levels and factors associated with it among patients attending a dental teaching institute in Himachal Pradesh. *SRM Journal of Research in Dental Sciences*.

[30] Al-Khalifa K. (2015). Prevalence of dental anxiety in two major cities in the kingdom of Saudi Arabia. *Saudi Journal of Medicine and Medical Sciences*.

[31] Brady P., Dickinson C., Whelton H. (2012). Dental anxiety prevalence and surgery environment Factors: a questionnaire-based survey of attenders in Ireland. *SAAD Digest*.

[32] Kastner A. K., Flohr E. L., Pauli P., Wieser M. J. (2016). A scent of anxiety: olfactory context conditioning and its influence on social cues. *Chemical Senses*.

[33] Appukuttan D. (2016). Strategies to manage patients with dental anxiety and dental phobia: literature review. *Clinical, Cosmetic and Investigational Dentistry*.

[34] Al-Omari W. M., Al-Omiri M. K. (2009). Dental anxiety among university students and its correlation with their field of study. *Journal of Applied Oral Science*.

